# Restricting calcium currents is required for correct fiber type specification in skeletal muscle

**DOI:** 10.1242/dev.129676

**Published:** 2016-05-01

**Authors:** Nasreen Sultana, Beatrix Dienes, Ariane Benedetti, Petronel Tuluc, Peter Szentesi, Monika Sztretye, Johannes Rainer, Michael W. Hess, Christoph Schwarzer, Gerald J. Obermair, Laszlo Csernoch, Bernhard E. Flucher

**Affiliations:** 1 Department of Physiology and Medical Physics, Medical University Innsbruck, Innsbruck 6020, Austria; 2 Department of Physiology, Faculty of Medicine, University of Debrecen, Debrecen 4032, Hungary; 3 Department of Pharmacology, University of Innsbruck, Innsbruck 6020, Austria; 4 Division of Molecular Pathophysiology, Biocenter, Medical University Innsbruck, Innsbruck 6020, Austria; 5 Division of Histology and Embryology, Medical University Innsbruck, Innsbruck 6020, Austria; 6 Department of Pharmacology, Medical University Innsbruck, Innsbruck 6020, Austria

**Keywords:** Voltage-gated calcium channel, Skeletal muscle excitation-contraction coupling, Muscle fiber type specification, Mouse

## Abstract

Skeletal muscle excitation-contraction (EC) coupling is independent of calcium influx. In fact, alternative splicing of the voltage-gated calcium channel Ca_V_1.1 actively suppresses calcium currents in mature muscle. Whether this is necessary for normal development and function of muscle is not known. However, splicing defects that cause aberrant expression of the calcium-conducting developmental Ca_V_1.1e splice variant correlate with muscle weakness in myotonic dystrophy. Here, we deleted *Ca_V_1.1* (*Cacna1s*) exon 29 in mice. These mice displayed normal overall motor performance, although grip force and voluntary running were reduced. Continued expression of the developmental Ca_V_1.1e splice variant in adult mice caused increased calcium influx during EC coupling, altered calcium homeostasis, and spontaneous calcium sparklets in isolated muscle fibers. Contractile force was reduced and endurance enhanced. Key regulators of fiber type specification were dysregulated and the fiber type composition was shifted toward slower fibers. However, oxidative enzyme activity and mitochondrial content declined. These findings indicate that limiting calcium influx during skeletal muscle EC coupling is important for the secondary function of the calcium signal in the activity-dependent regulation of fiber type composition and to prevent muscle disease.

## Abstract (precis)

**Summary:** Developmental splicing of Ca_V_1.1 restricts calcium currents in skeletal muscle, which are critical for activity-dependent regulation of fiber type specification and the prevention of mitochondrial damage.

## INTRODUCTION

Calcium is the principal second messenger regulating skeletal muscle contraction, growth and differentiation. In excitation-contraction (EC) coupling, cytoplasmic calcium levels are rapidly increased in response to action potentials and the magnitude of these calcium signals regulates the force of contraction. In skeletal muscle, voltage-gated calcium channels (Ca_V_1.1) and calcium release channels (type 1 ryanodine receptors, RyR1) are physically coupled to one another so that voltage-dependent activation of Ca_V_1.1 can directly activate opening of the RyR1. In mature muscles, calcium is released from the sarcoplasmic reticulum (SR) calcium stores, whereas calcium influx is dispensable for skeletal muscle EC coupling (Melzer et al., 1995). Actually, calcium currents through the major Ca_V_1.1a splice variant are small and activate slowly, only at strong membrane depolarizations.

Interestingly, during maturation of mammalian skeletal muscles activity-dependent calcium influx is actively suppressed by alternative splicing of Ca_V_1.1. In fetal muscles, exclusion of exon 29 produces a Ca_V_1.1e channel variant that conducts sizable L-type calcium currents and activates in parallel to SR calcium release at physiological voltages (Tuluc et al., 2009). However, after birth, the developmental Ca_V_1.1e splice variant is almost completely replaced by the adult, poorly conducting Ca_V_1.1a splice variant that includes exon 29 (Flucher and Tuluc, 2011; Tang et al., 2012). Why calcium influx is present in developing muscle but is then curtailed in mature skeletal muscles is not known. Conversely, it remains to be determined whether continued expression of the calcium-conducting Ca_V_1.1e splice variant alters contractile properties of mature skeletal muscles.

In addition to their primary role in EC coupling, activity-induced calcium signals in skeletal muscle are important for maintaining calcium homeostasis and for the regulation of muscle growth and differentiation. For example, calcium signals regulate the transcription of genes involved in the adaptive response to exercise (Bassel-Duby and Olson, 2006). Therefore, the tight control of calcium influx by alternative splicing of the Ca_V_1.1 channel is probably important for tuning muscle function to varying activity levels. Conversely, calcium influx through Ca_V_1.1e channels in mature muscles might be harmful. Abnormal expression of the embryonic, calcium-conducting Ca_V_1.1e splice variant in myotonic dystrophy type 1 (DM1) patients correlates with their degree of muscle weakness (Tang et al., 2012). Moreover, aberrant splicing of calcium channels and transporters in cultured myotubes from DM1 patients leads to altered intracellular calcium signaling (Santoro et al., 2014), and experimentally induced skipping of exon 29 aggravated the disease phenotype in muscles of a myotonia mouse model (Tang et al., 2012).

To identify the physiological importance of curtailing calcium influx through Ca_V_1.1 channels in adult skeletal muscle and to reveal a possible involvement of aberrant calcium signaling in DM1, we generated a genetic mouse model in which exon 29 has been permanently deleted. As expected, skeletal muscles of *Ca_V_1.1^ΔE29/ΔE29^* knockout mice experienced increased calcium influx during EC coupling and at rest. In addition, their contractile properties were altered, calcium-activated downstream regulators were upregulated, and the fiber type composition was shifted towards slower fiber types. However, mitochondrial content and oxidative enzyme activity were reduced. Together, these findings indicate that chronically increased calcium influx through the developmental Ca_V_1.1e isoform has little effect on EC coupling, but disturbs the normal regulation of muscle fiber type composition. Furthermore, the increased calcium influx causes mitochondrial damage and may thus contribute to muscle wasting in DM1. Conversely, these results suggest that, during normal development, limiting L-type calcium currents is important to enable the proper specification of fiber type composition and to protect the muscles from calcium-induced damage.

## RESULTS

### Selective deletion of *Ca_V_1.1* exon 29 prevents the developmental switch from the Ca_V_1.1e to the Ca_V_1.1a isoform

**Fig. 1.**
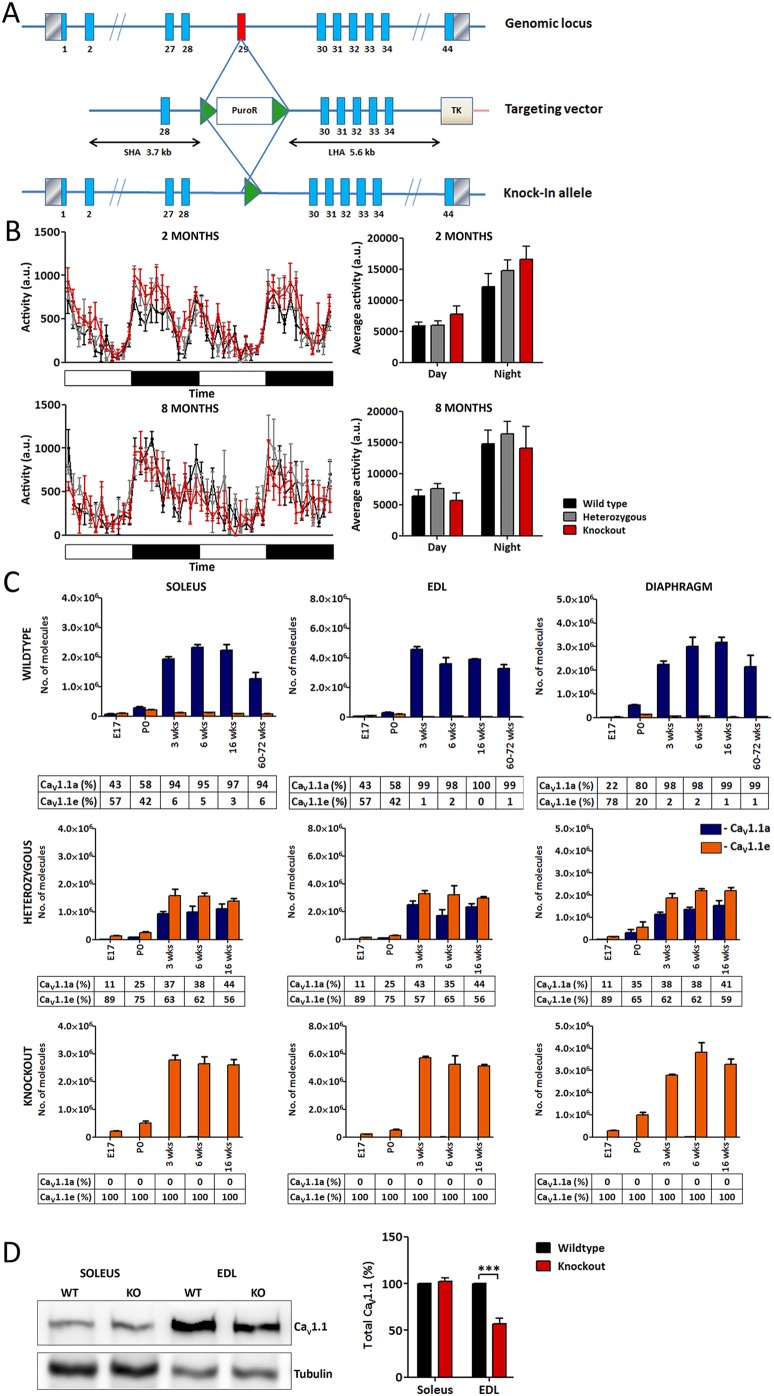
**Characterization of the *Ca_V_1.1^ΔE29^* mouse.** (A) Targeting strategy for generating the *Ca_V_1.1* exon 29 knockout allele. (B) Voluntary home cage activity at 2 and 8 months of age is similar in *Ca_V_1.1^ΔE29/ΔE29^* mice compared with wild-type and *Ca_V_1.1^+/ΔE29^* siblings (*N*=5). (C) Expression levels of *Ca_V_1.1a* and *Ca_V_1.1e* mRNAs in wild-type, *Ca_V_1.1^+/ΔE29^* and *Ca_V_1.1^ΔE29/ΔE29^* mice were measured by quantitative RT-PCR (TaqMan) in soleus, EDL and diaphragm muscle at different developmental stages (*N*=3). Numbers beneath show the fractional content of the two *Ca_V_1.1* transcripts. (D) Western blot analysis of total Ca_V_1.1 protein (both splice variants) in soleus and EDL muscle of wild-type and *Ca_V_1.1^ΔE29^* mice (*N*=3; ***P<0.001). Mean±s.e.m. See also Fig. S1.

In order to study the importance of the isoform switch from the calcium-conducting developmental Ca_V_1.1e splice variant to the poorly conducting adult Ca_V_1.1a splice variant we generated a mouse model with a constitutive knockout of exon 29 of the *Ca_V_1.1* (*Cacna1s*) gene (Fig. 1A). We reasoned that because the short transcript *Ca_V_1.1e* is predominant during fetal development, *Ca_V_1.1^ΔE29^* mice would develop normally up to birth, but that the aberrant continuing expression of the high-conductance developmental calcium channel splice variant throughout postnatal development and adult life would reveal any influence of the extra calcium influx on EC coupling and/or other calcium-mediated signaling processes regulating muscle growth and differentiation. Furthermore, the *Ca_V_1.1^ΔE29^* mouse will expose whether aberrant splicing of Ca_V_1.1 is itself sufficient to cause a disease phenotype reminiscent of DM1.

Heterozygous *Ca_V_1.1^+/ΔE29^* and homozygous *Ca_V_1.1^ΔE29/ΔE29^* mice were viable, they developed normally, and home cage activity of *Ca_V_1.1^ΔE29^* mice was not significantly different from that of wild-type siblings (Fig. 1B, Fig. S1A). Expression of the two *Ca_V_1.1* transcripts at different developmental stages was analyzed in the predominantly slow/oxidative soleus muscle, the predominantly fast/glycolytic extensor digitorum longus (EDL) muscle, and the mixed diaphragm muscle. Quantitative RT-PCR demonstrated that wild-type fetal muscles express moderate levels of both splice variants, with a higher proportion of the splice variant lacking exon 29 (Ca_V_1.1e) (Fig. 1C). After birth, wild-type muscles experienced a strong upregulation of the *Ca_V_1.1a* transcript, whereas expression of the *Ca_V_1.1e* transcript declined to less than 3% in 16-week-old mice. In muscles of ageing mice (15-18 months), total *Ca_V_1.1* transcript levels declined but the overall predominance of the Ca_V_1.1a variant was maintained. As expected, in homozygous *Ca_V_1.1^ΔE29/ΔE29^* mice the *Ca_V_1.1e* transcript was found exclusively. At all developmental stages its expression levels resembled those of total *Ca_V_1.1* transcripts in wild-type mice. Western blot analysis confirmed normal expression levels of total Ca_V_1.1 protein in soleus (Fig. 1D). However, in EDL muscle total Ca_V_1.1 protein was reduced. This might, at least in part, reflect a reduced content of triad junctions due to the fiber type shift observed in *Ca_V_1.1^ΔE29^* muscles (see below).

### Aberrant expression of the developmental Ca_V_1.1e isoform in mature muscles is not sufficient to cause severe myotonic dystrophy symptoms in mice

**Fig. 2.**
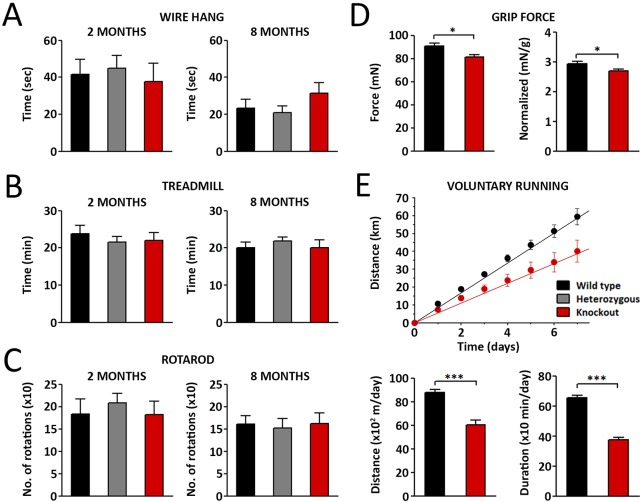
***Ca_V_1.1^ΔE29^* mice show normal motor performance but reduced muscle strength.** (A-C) Strength, endurance and motor skills were examined in wild-type, *Ca_V_1.1^+/ΔE29^* and *Ca_V_1.1^ΔE29/ΔE29^* mice using the wire hang test (A), treadmill running to exhaustion (B) and the Rotarod test (C). At 2 and 8 months of age, the performance of *Ca_V_1.1^ΔE29^* mice did not differ from that of the other genotypes (*N*=5-8; *P*>0.05). (D) Direct measurement of front paw strength revealed reduced grip force (both in absolute value and force normalized to body weight) in 6-month-old *Ca_V_1.1^ΔE29/ΔE29^* mice compared with wild-type siblings (*N*=5-8; *P<0.05). (E) Analysis of voluntary wheel running over a period of 7 days showed that the running distance and duration per day were decreased in *Ca_V_1.1^ΔE29/ΔE29^* mice compared with the wild type (*N*=8; ***P<0.001). Mean±s.e.m.

Because aberrant expression of Ca_V_1.1e in adults has been linked to the DM1 phenotype in mouse and human (Santoro et al., 2014; Tang et al., 2012), we subjected *Ca_V_1.1^ΔE29^* mice at 2 and 8 months of age to a range of behavioral tests to examine different aspects of muscle performance (Fig. 2). A wire hang test was used to assess muscle strength. Endurance was tested by making the mice run on a treadmill at accelerating speed until exhaustion. Overall motor performance was examined with the Rotarod test. In none of these tests was a significant difference in the performance of wild-type, heterozygous *Ca_V_1.1^+/ΔE29^* and homozygous *Ca_V_1.1^ΔE29/ΔE29^* mice observed. However, directly measuring grip strength revealed that the grip force of the front paws was significantly reduced in homozygous *Ca_V_1.1^ΔE29/ΔE29^* mice (Fig. 2D). Finally, voluntary running of the mice in a running wheel was recorded over the period of 7 days. Both the distance run and the duration the mice spent running per day were significantly reduced in homozygous *Ca_V_1.1^ΔE29/ΔE29^* mice compared with wild-type controls (Fig. 2E). These behavioral and functional analyses indicate that aberrant expression of Ca_V_1.1e alters muscle performance without causing severe motor deficits as assessed in tests that revealed the disease phenotype in other DM mouse models (Gomes-Pereira et al., 2011). Also, histological staining of muscle sections did not reveal an increase in centrally located nuclei in *Ca_V_1.1^ΔE29/ΔE29^* muscles (Fig. S1B).

### Aberrant expression of the developmental Ca_V_1.1e isoform in mature muscles alters the contractile properties of isolated slow and fast muscles

**Fig. 3.**
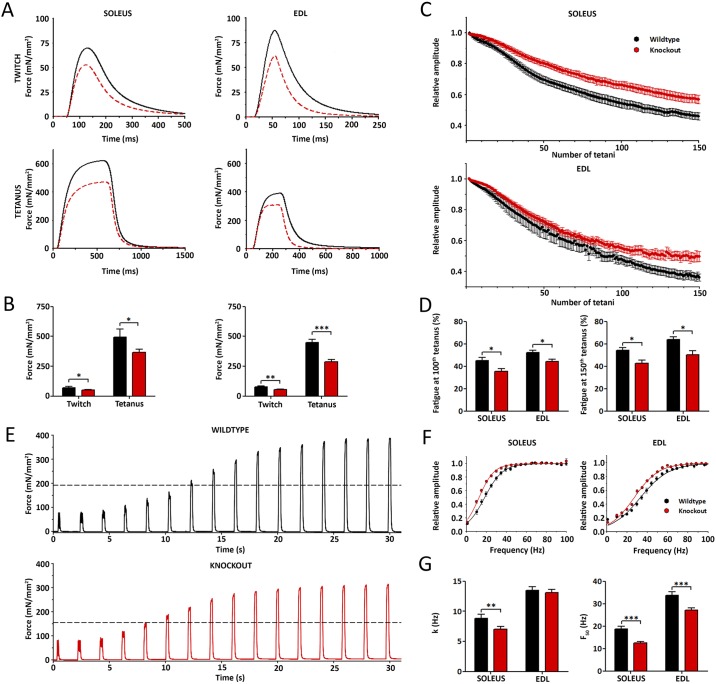
**Contractile properties of isolated slow and fast skeletal muscles are altered in *Ca_V_1.1^ΔE29/ΔE29^* mice.** (A) Representative recordings of twitch and tetanic contractions in isolated soleus and EDL muscles of 3- to 6-month-old wild-type (black; *N*=5) and *Ca_V_1.1^ΔE29/ΔE29^* (red; *N*=15) mice. Muscle twitches were elicited by a single 2 ms supramaximal electrical pulse (top), tetani by stimulation trains at 100 Hz for 500 ms in soleus (lower left) or at 200 Hz for 200 ms in EDL (lower right). (B) Maximal twitch and tetanic forces are significantly reduced in *Ca_V_1.1^ΔE29/ΔE29^* compared with control muscles (control *N*=5, *Ca_V_1.1^ΔE29/ΔE29^* *N*=15). (C) Decline of maximal force during 150 repetitive tetani in soleus (upper) and EDL (lower) muscles of wild-type (black) and *Ca_V_1.1^ΔE29/ΔE29^* (red) mice (stimulation as in A, repeated at 0.5 Hz; normalized to the first tetanus; control *N*=5, *Ca_V_1.1^ΔE29/ΔE29^* *N*=15). (D) Relative reduction of force at the 100th and 150th tetanus was significantly lower in *Ca_V_1.1^ΔE29/ΔE29^* than in wild-type muscles. (E) Representative force transients of isolated EDL muscle stimulated with increasing frequencies (10 to 85 Hz, with 0.5 Hz increment) from wild-type (black) and *Ca_V_1.1^ΔE29/ΔE29^* (red) mice. Note that 50% of maximal force (dashed line) is reached at 40 Hz (seventh transient) in wild-type and at 30 Hz (fifth transient) in *Ca_V_1.1^ΔE29/ΔE29^* muscles. (F) Relative force-frequency curves are left shifted in soleus (left) and EDL (right) muscles of *Ca_V_1.1^ΔE29/ΔE29^* (red) compared with wild-type (black) mice. (G) The frequency required for producing half-maximal force was significantly lower in soleus and EDL muscles of *Ca_V_1.1^ΔE29/ΔE29^* compared with wild type (control *N*=7, *Ca_V_1.1^ΔE29/ΔE29^* *N*=12). *P<0.05, **P<0.01, ***P<0.001. Mean±s.e.m.

Because the reduced grip force and voluntary running indicated altered muscle performance, we next determined the contractile properties directly in isolated mouse muscles. The contractile force of soleus and EDL muscles was recorded in response to a single electrical stimulus (twitch) and in response to high-frequency trains of stimuli (tetanus) (Fig. 3A). Both were significantly reduced in both muscles types of *Ca_V_1.1^ΔE29/ΔE29^* mice (Fig. 3B, Table 1). The relative decline of muscle force during a continuous series of repetitive tetanic stimulations is a measure of the fatigability of the muscle (Fig. 3C). Slow soleus muscles are more fatigue resistant than fast EDL muscles. In *Ca_V_1.1^ΔE29/ΔE29^* mice, both muscle types display significantly reduced fatigue compared with wild-type controls (Fig. 3D, Table 1). Finally the tetanic fusion frequency was assessed by recording tetanic force during a series of stimulus trains of increasing frequency (Fig. 3E). *Ca_V_1.1^ΔE29/ΔE29^* muscles reached the half-maximal value and the plateau of contractile force at lower frequencies than control muscles. This is reflected by a left shift of the force frequency curve (Fig. 3F) and a reduction of the frequency of the half-maximal tetanic force (Fig. 3G) in both soleus and EDL muscles. Together, these tests on isolated muscles demonstrate that expression of Ca_V_1.1e in adult *Ca_V_1.1^ΔE29/ΔE29^* mice significantly reduces the contractile force, increases the fatigue resistance, and lowers the tetanic fusion frequency of slow and fast muscles.

**Table 1.**
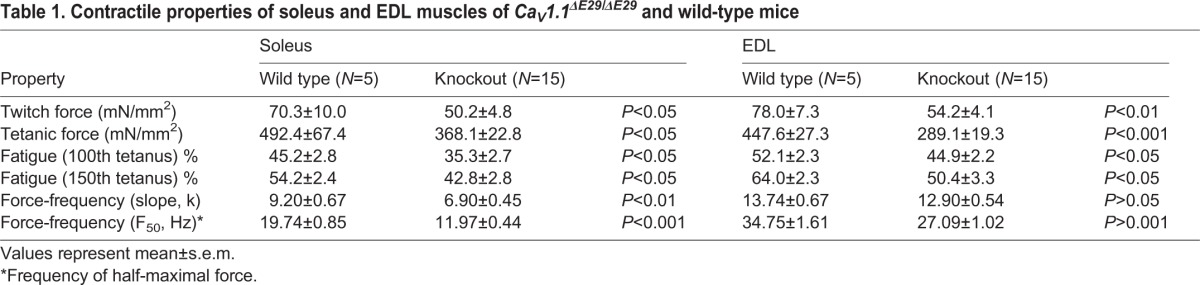
**Contractile properties of soleus and EDL muscles of *Ca_V_1.1^ΔE29/ΔE29^* and wild-type mice**

### Aberrant expression of the developmental Ca_V_1.1e isoform in mature muscles alters calcium signals during EC coupling and in resting muscle fibers

**Fig. 4.**
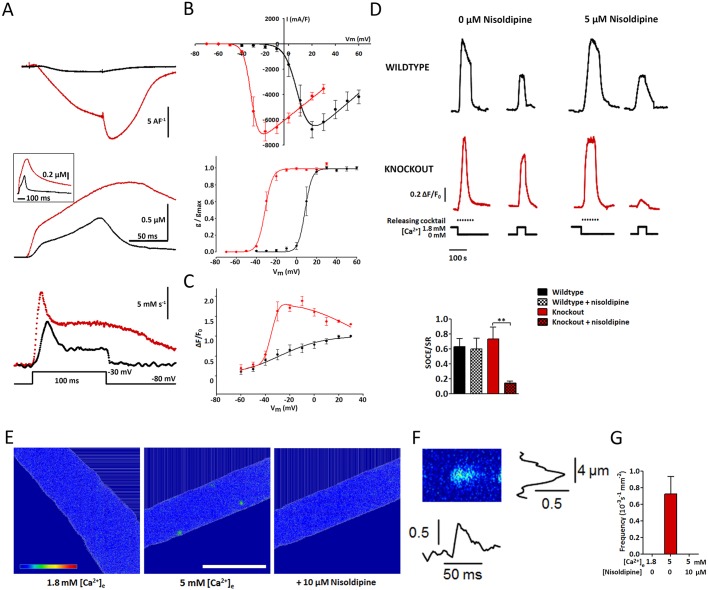
**Altered calcium signaling in isolated muscle fibers of *Ca_V_1.1^ΔE29/ΔE29^* mice.** Enzymatically isolated FDB fibers from 3- to 4-month-old *Ca_V_1.1^ΔE29/ΔE29^* (red) and wild-type (black) mice were loaded with Rhod-2 (A-C) or Fluo-8 AM (D-G). (A) Representative voltage-clamp recording of calcium currents and parallel recording of cytoplasmic free calcium during a 100 ms depolarization to −30 mV. Bottom trace is the calculated total calcium flux. (B) Voltage dependence of peak current densities and conductance display the 38.5±3.1 mV shift of channel activation in *Ca_V_1.1^ΔE29/ΔE29^* fibers. Note that *Ca_V_1.1^ΔE29/ΔE29^* fibers were recorded in 1.8 mM extracellular calcium during 100 ms test pulses and control fibers in 5 mM calcium during 500 ms test pulses to experimentally normalize current densities. (C) Calcium transient amplitudes display a striking increase in *Ca_V_1.1^ΔE29/ΔE29^* muscle fibers at intermediate voltages, indicative of the calcium influx through Ca_V_1.1e. (D) Representative calcium recordings of wild-type and *Ca_V_1.1^ΔE29/ΔE29^* fibers during an SR calcium release and reloading protocol. Note that the relative magnitude of the release and reloading transients is significantly decreased upon nisoldipine block of Ca_V_1 channels in *Ca_V_1.1^ΔE29/ΔE29^* but not in wild-type muscle (**P<0.01). (E) Representative confocal images of a resting *Ca_V_1.1^ΔE29/ΔE29^* FDB fiber in 1.8 mM and 5 mM calcium. Spontaneous calcium release events occur only in 5 mM calcium and are blocked by addition of 10 µM nisoldipine. (F) Spatiotemporal properties in line-scan images identify the localized calcium signals as calcium sparklets. (G) The sparklets are sensitive to the extracellular calcium concentration and L-type channel block. *n*=32 images/*N*=4 animals (1.8 mM), *n*=183/*N*=7 (5 mM) and *n*=24/*N*=3 (5 mM+nisoldipine). Mean±s.e.m. See also Fig. S2.

To clarify the cellular mechanisms underlying the altered muscle properties of *Ca_V_1.1^ΔE29/ΔE29^* mice we analyzed calcium currents and cytoplasmic calcium signals directly in isolated flexor digitorum brevis (FDB) muscle fibers using several experimental paradigms. First, combined patch-clamp and cytoplasmic calcium recording was performed in FDB fibers loaded with the fluorescent calcium indicator Rhod-2. In line with the current properties of the Ca_V_1.1e splice variant previously determined in reconstituted dysgenic myotubes (Tuluc et al., 2009), *Ca_V_1.1^ΔE29/ΔE29^* FDB fibers displayed sizable calcium currents starting at test potentials of −30 mV (Fig. 4A). Under the same conditions (1.8 mM extracellular calcium, 100 ms test pulses), control FDB fibers did not display measurable currents at any test potential. In order to compare voltage sensitivity between the two genotypes, recordings in control fibers were repeated using 5 mM extracellular calcium and 500 ms test pulses. The current-voltage and current-conductance curves indicate that in *Ca_V_1.1^ΔE29/ΔE29^* fibers half-maximal current activation is shifted by 38.5±3.1 mV in the hyperpolarizing direction (Fig. 4B). Accordingly, the simultaneously recorded calcium transients in *Ca_V_1.1^ΔE29/ΔE29^* fibers showed a pronounced voltage-dependent component that peaked at −20 mV and declined in parallel with the current density at positive potentials (Fig. 4C).

From the calcium transients the total calcium flux (influx and SR release) during the depolarizing pulses was calculated (Fig. 4A lower trace, Fig. S2). These calcium flux traces characteristically showed an early peak followed by a steady-state plateau phase (Szentesi et al., 1997). In control muscle, the voltage dependence of both the peak and plateau calcium fluxes displayed a monotonic increase, which could be fitted with a two-state Boltzmann function. This is consistent with a model of skeletal muscle EC coupling in which both parameters of the calcium flux (peak and plateau) represent a single process, i.e. calcium release from the SR. In *Ca_V_1.1^ΔE29/ΔE29^* muscle fibers, the peak calcium flux behaved like that in controls, whereas the voltage dependence of the plateau flux was non-monotonous. At intermediate voltages the plateau flux was significantly larger than that of controls. Thus, the additional component in *Ca_V_1.1^ΔE29/ΔE29^* muscles compared with controls corresponds to voltage-dependent calcium influx, which peaks at −20 mV where most Ca_V_1.1e channels are activated and then decreases in parallel with the declining driving force at positive membrane potentials. Together, these analyses clearly demonstrate that, during EC coupling, muscle fibers of *Ca_V_1.1^ΔE29/ΔE29^* mice experience increased calcium signals due to a substantial component of calcium influx that is not observed in muscle fibers of wild-type mice.

In addition to its immediate role in EC coupling, this extra calcium influx in *Ca_V_1.1^ΔE29/ΔE29^* mice might alter the calcium homeostasis in muscle cells. Analysis of cytoplasmic calcium concentrations in Fura-2-loaded isolated FDB fibers revealed no difference in resting calcium levels of *Ca_V_1.1^ΔE29/ΔE29^* mice compared with wild-type controls (45.77±0.96 nM and 46.14±1.14 nM, respectively; *P*>0.5). Next, we examined a possible contribution of L-type calcium currents to refilling of SR calcium stores. Both SR depletion and refilling resulted in a robust cytoplasmic calcium transient (Fig. 4D), the relative magnitude of which was expressed as the external to SR calcium transient ratio. In wild-type muscle fibers this ratio was not affected by the application of the L-type calcium channel blocker nisoldipine. However, in *Ca_V_1.1^ΔE29/ΔE29^* muscle fibers 5 µM nisoldipine dramatically reduced the ratio, indicating that in Ca_V_1.1e-expressing muscle SR refilling is predominantly carried out by calcium influx through L-type channels (i.e. Ca_V_1.1e).

Finally, the spontaneous occurrence of focal calcium transients – so called calcium sparklets – in resting muscle fibers was investigated. Because similar local calcium release events are exclusive to muscle cells expressing calcium-conducting Ca_V_ channels (e.g. Ca_V_1.2 in cardiac myocytes), we hypothesized that aberrant expression of Ca_V_1.1e might also bring about calcium sparklets in mature muscle fibers of *Ca_V_1.1^ΔE29/ΔE29^* mice. Indeed, isolated FDB fibers from *Ca_V_1.1^ΔE29/ΔE29^* mice loaded with Fluo-8 AM displayed spontaneous calcium sparklet-like behavior (Fig. 4E). The average amplitude of these calcium sparklets was 0.140±0.001 [ΔF/F]. The average amplitude, full width and half maxima of these calcium sparklets resembled those described by Rodríguez et al. (2014). In 5 mM extracellular calcium, these calcium sparklets occurred at a frequency of 0.724±0.211 10^−3^ s^−1^ mm^−1^. When the extracellular calcium concentration was reduced to 1.8 mM, or when L-type calcium channels were blocked with 10 µM nisoldipine, the calcium sparklets were completely abolished, indicating their dependence on calcium influx through Ca_V_1.1e. In wild-type control muscle fibers no such calcium release events were observed in normal or high extracellular calcium concentrations. Together, these findings demonstrate that calcium influx through the Ca_V_1.1e splice variant not only alters calcium handling during EC coupling and during refilling of SR calcium stores, but also causes spontaneous calcium signals in resting intact muscle fibers.

### Muscles of Ca_V_1.1e-expressing mice display an altered fiber type composition and oxidative metabolism

In skeletal muscle, calcium signals also regulate activity-dependent control of muscle growth and fiber type specification. Moreover, the observed changes in contractile properties – decreased force, increased fatigue resistance and lower tetanic fusion frequency – are all reminiscent of the differences between fast and slow muscle types. Therefore, we hypothesized that the altered calcium signaling in *Ca_V_1.1^ΔE29/ΔE29^* mice might affect contractile properties indirectly by a dysregulation of fiber type specification.

**Fig. 5.**
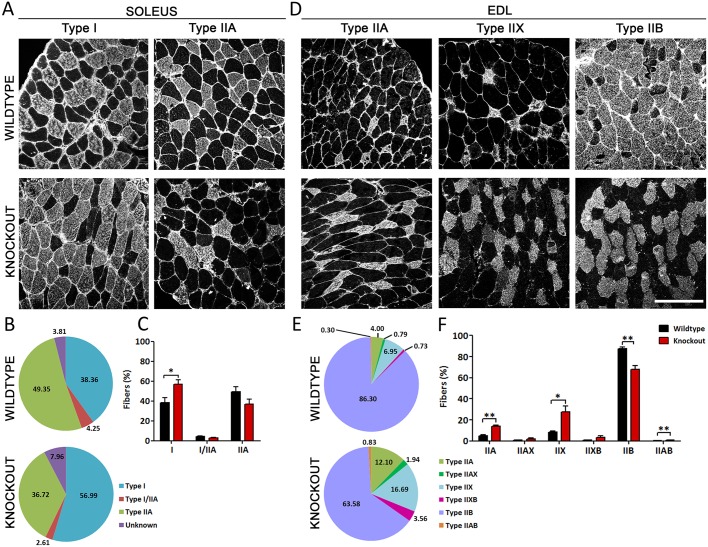
**Changes in fiber type composition and metabolic properties of soleus and EDL muscles in *Ca_V_1.1^ΔE29/ΔE29^* mice.** (A,D) Representative transverse sections of soleus and EDL muscles from 5- to 6-month-old wild-type and *Ca_V_1.1^ΔE29/ΔE29^* mice immunostained with fiber type-specific myosin heavy chain antibodies. Scale bar: 100 µm. Note the increase of type I fibers and decrease of type IIA fibers in *Ca_V_1.1^ΔE29/ΔE29^* soleus (A), and the increase of IIA and IIX fibers in parallel with the decrease of IIB fibers in *Ca_V_1.1^ΔE29/ΔE29^* EDL (D). (B,E) The fractional redistribution of fiber types in *Ca_V_1.1^ΔE29/ΔE29^* soleus and EDL muscles. (C,F) The relative magnitude and statistical significance of the changes in individual fiber types. *N*=3; *P<0.05, **P<0.01. Mean±s.e.m.

To analyze the fiber type composition of slow and fast muscles in wild-type and *Ca_V_1.1^ΔE29/ΔE29^* mice, we immunostained sections of soleus and EDL muscle with antibodies against specific myosin heavy chain isoforms. The representative images in Fig. 5A,D demonstrate a substantial shift towards slower fiber types in both soleus and EDL muscles of *Ca_V_1.1^ΔE29/ΔE29^* mice. Soleus muscles of *Ca_V_1.1^ΔE29/ΔE29^* mice experienced a 48% increase in the fraction of type I fibers, mainly at the cost of type IIA and mixed fibers (Fig. 5A-C). In EDL muscle of *Ca_V_1.1^ΔE29/ΔE29^* mice the fraction of type IIB fibers was reduced by 26%, whereas the fractions of IIA, IIX and mixed fibers increased 2- to 3-fold (Fig. 5D-F). Type I fibers were not detected in *Ca_V_1.1^ΔE29/ΔE29^* EDL muscles. These findings indicate that expression of the calcium-conducting Ca_V_1.1e splice variant in skeletal muscles of adult *Ca_V_1.1^ΔE29/ΔE29^* mice causes a substantial shift in fiber type composition towards slower fiber types.

### Aberrant expression of Ca_V_1.1e in mature skeletal muscle causes mitochondrial damage

**Fig. 6.**
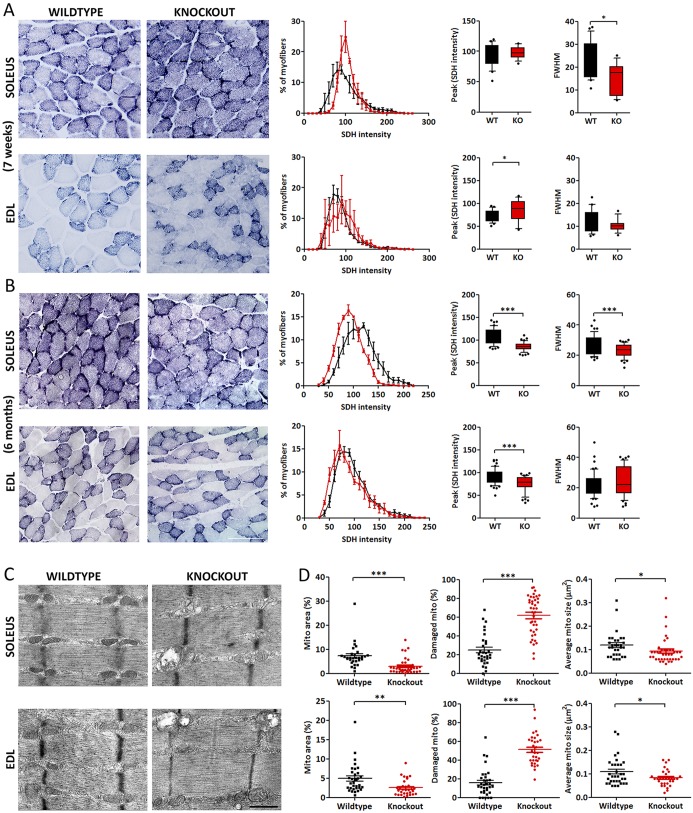
**Mitochondrial content and enzyme activity are reduced in *Ca_V_1.1^ΔE29/ΔE29^* muscles.** (A,B) SDH activity was analyzed in sections of wild-type (black) and *Ca_V_1.1^ΔE29/ΔE29^* (red) mice. Staining intensity was measured in each fiber profile and plotted in intensity distribution diagrams. (A) In 7-week-old mice, SDH activity is markedly increased in *Ca_V_1.1^ΔE29/ΔE29^* soleus and EDL muscles, as seen by the right-shifted intensity distribution histogram. (B) In 6-month-old mice, SDH activity is significantly reduced in *Ca_V_1.1^ΔE29/ΔE29^* soleus muscle and in EDL muscles. In EDL also the full width at half maximum (FWHM) was reduced. *N*=3. (C) Electron micrographs of 4- and 5-month-old wild-type and *Ca_V_1.1^ΔE29/ΔE29^* soleus and EDL muscles. Whereas myofibrils and EC coupling membranes appear normal, dilated and lysed mitochondria are found in *Ca_V_1.1^ΔE29/ΔE29^* muscles. (D) Morphometric analysis demonstrates significantly decreased fractional content (percentage area occupied by intact mitochondria) in both *Ca_V_1.1^ΔE29/ΔE29^* muscle types. Mitochondrial size is decreased in *Ca_V_1.1^ΔE29/ΔE29^* muscles and the fraction of damaged mitochondria increased in *Ca_V_1.1^ΔE29/ΔE29^* soleus muscles. *N*=30-40 images of two biological replicates per condition. *P<0.05, **P<0.01, ***P<0.001. Mean±s.e.m. See also Fig. S3. Scale bars: 100 µm in A,B; 0.5 µm in C.

A slower fiber type composition is expected to be accompanied by an increase in oxidative metabolism. Indeed, staining of succinate dehydrogenase (SDH) in soleus and EDL muscles of 7-week-old *Ca_V_1.1^ΔE29/ΔE29^* mice revealed a marked increase in SDH activity compared with wild-type controls (Fig. 6A). In soleus, a loss of fibers with lower SDH activity was evident, probably reflecting the increase in type I fibers relative to type IIA fibers. In EDL, an increase in high SDH activity fibers occurred, consistent with the increased fraction of oxidative IIA fibers relative to glycolytic fiber types (see Fig. 5). Unexpectedly, in 6- and 12-month-old *Ca_V_1.1^ΔE29/ΔE29^* mice the SDH activity was significantly reduced compared with the wild type (Fig. 6B, Fig. S3A). The left shift of the intensity distribution diagrams was more pronounced in soleus, but it was still significant in EDL. It equally affected fibers with low and high SDH activity. Together, these results show that the initial increase of oxidative metabolism in young *Ca_V_1.1^ΔE29/ΔE29^* mice is lost and even reversed at 6 months and older.

Because decreased SDH activity could arise from either a reduction in mitochondrial activity or content, we analyzed mitochondria in electron microscopy preparations. Consistent with the overall healthy state and normal motor performance of the *Ca_V_1.1^ΔE29/ΔE29^* mice, electron microscopy did not reveal any defects in the myofibrils and EC coupling membranes (Fig. 6C). However, the mitochondria were distorted in *Ca_V_1.1^ΔE29/ΔE29^* muscles. Morphometric analysis revealed that the mitochondrial content was significantly reduced in *Ca_V_1.1^ΔE29/ΔE29^* mice to approximately half that in wild-type controls (Fig. 6D, Fig. S3B). This loss of intact mitochondria was paralleled by an increase in the fraction of damaged mitochondria up to 4.5-fold compared with wild-type controls, as well as a decrease in the average size of the healthy mitochondria. These findings explain the significantly reduced SDH activity observed in *Ca_V_1.1^ΔE29/ΔE29^* compared with wild-type muscles of the same age.

### Aberrant expression of Ca_V_1.1e changes key activity- and calcium-dependent regulators of fiber type specification and mitochondrial biogenesis

**Fig. 7.**
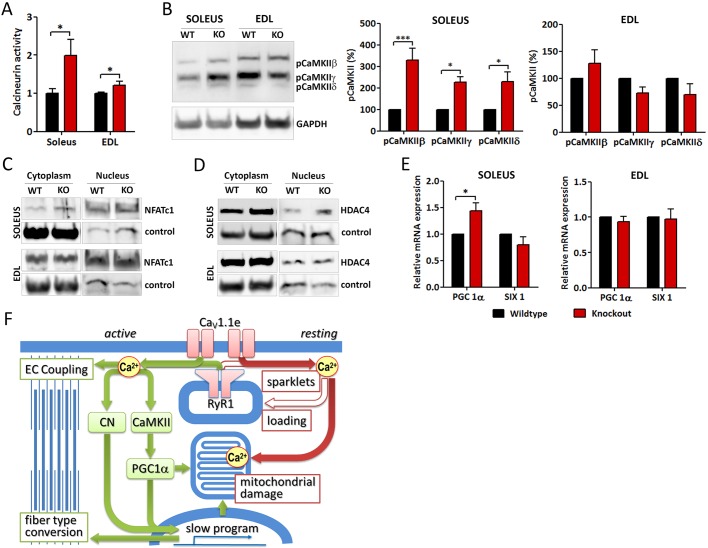
**Altered expression of regulators of fiber type specification and mitochondrial biogenesis in slow and fast muscles of *Ca_V_1.1^ΔE29/ΔE29^*** **mice.** (A) Basal enzymatic activity of calcineurin is significantly increased in *Ca_V_1.1^ΔE29/ΔE29^* soleus and EDL muscles (red) compared with wild type (black). (B) Western blot analysis shows a significant increase of activated CaMKII β, γ and δ isoforms (phosphorylated at Thr286) in soleus but not EDL muscle of *Ca_V_1.1^ΔE29/ΔE29^*. (C,D) Cytoplasmic and nuclear localization of NFATC1 and HDAC4 does not differ between the genotypes (age of mice 6-8 months). Controls for the cytoplasm and nuclear fraction are GAPDH and histone H3, respectively. *N*=3. (E) Quantitative RT-PCR analysis demonstrates that expression of *PGC1*α (*Ppargc1a*) mRNA is significantly increased in *Ca_V_1.1^ΔE29/ΔE29^* soleus muscles, whereas the decline of *PGC1*α in EDL and of *Six1* expression in both muscles is not significant. *N*=3. *P<0.05, ***P<0.001. Mean±s.e.m. (F) Model of signaling pathways causing fiber type shift and mitochondrial damage in *Ca_V_1.1^ΔE29/ΔE29^* muscles. During EC coupling, increased calcium influx through Ca_V_1.1e activates calcineurin (CN), CaMKII and PGC1α, the primary regulators of fiber type specification and mitochondrial biogenesis. The shift in fiber type composition in the slow direction offsets the direct effects of increased calcium signals on EC coupling. Parallel upregulation of oxidative metabolism by PGC1α is counteracted by mitochondrial damage caused by calcium overload due to increased activity-dependent and spontaneous calcium influx and altered calcium homeostasis. See also Fig. S4.

If the altered calcium signals in Ca_V_1.1e-expressing muscles impact the regulation of fiber type specification this might be reflected in the activity and/or expression levels of major calcium- and activity-regulated signaling proteins. In skeletal muscle, the calcium-dependent protein phosphatase calcineurin (protein phosphatase 2B) and the calmodulin-dependent protein kinase II (CaMKII) decode fiber type-specific activation patterns and function as master regulators of fast to slow fiber type changes (Chin et al., 1998; Wu et al., 2001; Chin, 2005). Using a colorimetric phosphatase assay we show that steady-state calcineurin activity is significantly increased in *Ca_V_1.1^ΔE29/ΔE29^* soleus and EDL muscles (Fig. 7A). Western blot analysis using a phospho-specific antibody demonstrated that the activated forms of all three CaMKII isoforms were significantly increased in cytoplasmic fractions of soleus muscle (Fig. 7B), whereas in EDL muscles no changes in CaMKII activation were observed. A differential activation of CaMKII in slow versus fast muscles is consistent with its suggested role in differentially decoding slow and fast muscle calcium signals (Tavi and Westerblad, 2011).

Interestingly, expression of their respective downstream transcriptional regulators, NFATC1 and HDAC4, in the cytoplasm and nuclei of *Ca_V_1.1^ΔE29/ΔE29^* muscles was not altered (Fig. 7C,D, Fig. S4A,B). However, expression of peroxisome proliferator-activated receptor γ co-activator 1α (*PGC1*α; *Ppargc1a*), a key downstream regulator of mitochondrial biogenesis and of oxidative metabolism in muscle (Handschin et al., 2007; Lin et al., 2002), was significantly increased in soleus muscle of *Ca_V_1.1^ΔE29/ΔE29^* mice compared with wild-type controls (Fig. 7E). In EDL muscle, *PGC1*α expression was not significantly altered. Expression of *Six1*, a key regulator of the fast fiber program (Grifone et al., 2004; Wu et al., 2013), was not affected.

To further examine potential effects on the expression patterns of the slow and fast program and on mitochondrial biogenesis, Affymetrix expression profiling was performed on mRNA preparations from soleus and EDL muscles of wild-type and *Ca_V_1.1^ΔE29/ΔE29^* mice. Although the analysis showed differential gene expression in soleus versus EDL muscles of both genotypes, comparison of wild-type versus *Ca_V_1.1^ΔE29/ΔE29^* soleus and EDL muscles did not reveal any significant differences (Fig. S4C). Also, the specific analysis of genes involved in mitochondrial fusion and fission revealed only small differences between slow and fast muscles but not between wild-type and *Ca_V_1.1^ΔE29/ΔE29^* mice. Thus, the increased calcium influx through Ca_V_1.1e chronically hyperactivates calcineurin, CaMKII and PGC1α signaling and, over time, produces the observed changes in fiber type composition without, however, a major induction of the slow muscle gene program at basal activity levels in adult mice.

## DISCUSSION

### The developmental switch of Ca_V_1.1 splice variants is important for the correct specification of skeletal muscle fiber types

Several lines of evidence at the molecular, functional and behavioral level demonstrate that the continued expression of the developmental Ca_V_1.1e splice variant in mature muscles of *Ca_V_1.1^ΔE29/ΔE29^* mice alters the fiber type composition in the slow direction: (1) in soleus muscle the fraction of type I fibers increases at the expense of type IIA fibers, and in EDL muscles type IIA, IIX and mixed fibers increase at the expense of type IIB fibers; (2) in isolated soleus and EDL muscles maximal twitch and tetanic forces are reduced, fatigue resistance is increased, and tetanic fusion of contractions occurs at lower frequencies; (3) grip force and the duration of voluntary wheel running are reduced; (4) calcineurin and CaMKII activity and expression of PGC1α are upregulated. However, in mature mice the increased slow fiber content and the increased expression of PGC1α were no longer accompanied by the expected increase in oxidative enzyme activity, most likely because the increased calcium influx in *Ca_V_1.1^ΔE29/ΔE29^* muscles also caused a severe loss of mitochondria.

The fiber type composition of skeletal muscles is primarily a genetically determined adaptation of muscle properties to their specific physiological functions. Furthermore, it is subject to continuous dynamic adaptation to altered demand. The changes in fiber type composition and contractile properties observed in the *Ca_V_1.1^ΔE29/ΔE29^* mice are reminiscent of adaptations occurring in response to endurance training. The magnitude of the observed shift in fiber type composition is within the range observed in mice subjected to stringent endurance training protocols (Allen et al., 2001; Krüger et al., 2013). However, neither monitoring home cage activity nor the behavioral tests revealed increased spontaneous activity. If anything, *Ca_V_1.1^ΔE29/ΔE29^* mice spent less time voluntarily running than wild-type controls. Therefore, the observed changes in fiber type composition cannot be explained as a normal adaptive response to altered activity, but represent a dysregulation of fiber type composition owing to the absence of postnatal inclusion of *Ca_V_1.1* exon 29 in *Ca_V_1.1^ΔE29/ΔE29^* mice. Since Ca_V_1.1 is almost exclusively expressed in skeletal muscle and the functional effects (e.g. reduced force, increased fatigue resistance) were observed in isolated muscles, muscle-intrinsic mechanisms are likely to be responsible for the altered muscle fiber type composition in *Ca_V_1.1^ΔE29/ΔE29^* mice.

### Expression of Ca_V_1.1e alters skeletal muscle calcium signaling and activates the pathways for the slow twitch fiber program

Calcium is the principal second messenger regulating adaptive changes of muscle properties in response to training or experimentally altered innervation patterns (Bassel-Duby and Olson, 2006; Chin et al., 1998; Liu et al., 2005). As the primary defects in the *Ca_V_1.1^ΔE29/ΔE29^* mice are altered gating and conduction properties of the skeletal muscle L-type calcium channel expressed in adult mice, a role of increased calcium influx in determining the fiber type composition is very likely. In cultured dysgenic myotubes reconstituted with either of two Ca_V_1.1 splice variants, we previously reported that exclusion of exon 29 caused a 30 mV left-shifted voltage dependence of current activation and an 8-fold increase in current density (Tuluc et al., 2009). Here, we demonstrate that also in isolated muscle fibers of *Ca_V_1.1^ΔE29/ΔE29^* mice, sole expression of the Ca_V_1.1e splice variant causes an equally large left shift of voltage sensitivity and a substantially increased calcium influx during EC coupling. Interestingly, the calcium influx on top of the calcium released from the SR was not reflected in a parallel increase in contractile strength. This indicates that, with respect to muscle contraction, the effects of increased calcium influx are counteracted by other consequences of the altered calcium signaling in *Ca_V_1.1^ΔE29/ΔE29^* mice. In fact, the observed fiber type shift to slower, and thus weaker, fiber types could be part of a compensatory response of the muscle cell to an increased calcium load during EC coupling. The effects of continued expression of the calcium-conducting Ca_V_1.1e splice variant on fatigability and fiber type composition are consistent with recently published results obtained in a *Ca_V_1.1* mutant (Lee et al., 2015). A knock-in mouse with a mutation in the Ca_V_1.1 pore that abolished calcium binding and conduction showed increased fatigability and type IIB fiber content. Together, the two studies demonstrate that fiber type specification in skeletal muscle is highly sensitive to the magnitude of L-type calcium currents. The loss of calcium influx through Ca_V_1.1 shifts the muscles towards a faster phenotype, whereas an increase of calcium currents causes a shift towards a slower phenotype.

In addition to altered calcium transients during EC coupling, a nisoldipine-sensitive component of calcium influx was observed in a store-refilling protocol, indicating that the extra calcium influx through the developmental Ca_V_1.1e splice variant also contributes to mechanisms of calcium homeostasis. Functionally, increased store filling probably contributes to the increased fatigue resistance observed in muscles of *Ca_V_1.1^ΔE29/ΔE29^*mice. Consistent with this notion, the opposite effects – decreased store filling and decreased fatigue resistance – were observed in the calcium permeation mutant mouse (Lee et al., 2015).

Furthermore, at rest muscle fibers of *Ca_V_1.1^ΔE29/ΔE29^* mice generated focal calcium transients – so called calcium sparklets. Although calcium sparks are common in cardiac muscle cells, in developing skeletal muscle fibers and in cultured myotubes, they have never before been observed in mature muscle fibers of healthy mice (Cheng and Lederer, 2008; Weisleder and Ma, 2006). The calcium sparklets observed in *Ca_V_1.1^ΔE29/ΔE29^* mice were dependent on the extracellular calcium concentration and were sensitive to the L-type channel blocker nisoldipine. Thus, there can be little doubt that calcium influx through Ca_V_1.1e channels contributed directly or indirectly to this phenomenon. As calcium sparks in cardiac myocytes can be activated by the opening of even a single Ca_V_1.2 channel (Lopez-Lopez et al., 1995), it is conceivable that in skeletal muscles spontaneous openings of Ca_V_1.1e channels trigger the opening of RyR1 and thus activate calcium sparklets by calcium-induced calcium release. In wild-type mice this is limited to developing myotubes, which naturally express the Ca_V_1.1e splice variant. In *Ca_V_1.1^ΔE29/ΔE29^* mice these spontaneous calcium signals occur also in differentiated muscles, where they might affect signaling pathways regulating fiber type composition and cause mitochondrial damage.

Downstream of calcium, it is calcineurin and CaMKII that are the key regulators of fast to slow twitch fiber type changes (Chin et al., 1998). Calcineurin regulates transcription of muscle genes via the classical NFAT pathway, CaMKII via HDAC4. Both converge on MEF2, but have also been reported to activate PGC1α (Tavi and Westerblad, 2011). A key feature of these parallel signaling pathways is their ability to distinguish between calcium signals in response to chronic, repetitive (slow fiber type) activation patterns and those in response to phasic (fast fiber type) activation patterns (Liu et al., 2005; Tavi and Westerblad, 2011). Apparently, expression of a calcium-conducting Ca_V_1.1e splice variant in adult muscles tips the balance of this delicate calcium sensing mechanism and leads to constitutive activation of the signaling pathway for the slow muscle program (Fig. 7F). Consistent with this notion, we observed that calcineurin and CaMKII activity, as well as expression of PGC1α, were constitutively upregulated in skeletal muscles of *Ca_V_1.1^ΔE29/ΔE29^* mice, and that activation of CaMKII and PGC1α was specific to slow muscles. In accordance with this, altered CaMKII signaling was also observed in the calcium permeation *Ca_V_1.1* mutant (Lee et al., 2015).

Thus, in *Ca_V_1.1^ΔE29/ΔE29^* mice the continuous expression of Ca_V_1.1e causes increased calcium influx during EC coupling, in homeostatic calcium regulation, and at rest. Which of these calcium influx events contribute to the activation of the slow muscle pathway remains to be determined. Because calcineurin and CaMKII signaling is highly sensitive to the calcium signaling patterns in response to slow fiber type-specific activity, we favor a role of altered calcium signal during EC coupling. In any case, if aberrant activation of these signaling pathways causes an increase in slow fibers in *Ca_V_1.1^ΔE29/ΔE29^* mice, during normal development the alternative splicing event causing the shift from a calcium-conducting to a non-conducting Ca_V_1.1 variant might be an important prerequisite for the proper regulation of fiber type composition at basal activity levels as well as in response to exercise.

### The contribution of aberrant Ca_V_1.1 splicing to myotonic dystrophy

Splicing defects of important muscle proteins, including the CLCN1 chloride channel, insulin receptor, SERCA1 (ATP2A1) and Ca_V_1.1, lead to DM1 (Thornton, 2014). The myotonia is likely to be caused by a hyperexcitability of muscles due to the loss of CLCN1 function (Lueck et al., 2007). Because aberrant expression of the Ca_V_1.1e splice variant correlates with the degree of muscle weakness in DM1 patients, and forced missplicing of *Ca_V_1.1* exon 29 caused centrally localized nuclei in a myotonia mouse model, it has been suggested that increased calcium influx through the developmental Ca_V_1.1e splice variant may contribute to the myopathy (Santoro et al., 2014; Tang et al., 2012). This hypothesis is in line with the known role of increased calcium influx via various entry pathways in muscular dystrophy (Allen et al., 2010; Whitehead et al., 2006). Here, we examined whether aberrant missplicing of *Ca_V_1.1* exon 29 by itself is sufficient to cause DM-like symptoms. At the organismal level this was not the case. Homozygous *Ca_V_1.1^ΔE29/ΔE29^* mice, which exclusively express the developmental Ca_V_1.1e splice variant, did not show severe muscle weakness, and their muscle sections did not reveal centrally located nuclei, which is a histopathological hallmark of dystrophic muscle. Although the contractile force of isolated muscles was reduced, this was accompanied by increased fatigue resistance and might therefore be the consequence of the fiber type shift rather than a symptom of DM1.

We observed decreased SDH activity and severe mitochondrial damage in muscles of mature and aged *Ca_V_1.1^ΔE29/ΔE29^* mice. Similar mitochondrial damage has been described in other mouse muscle disease models with aberrant calcium signaling (Irwin et al., 2003). Calcium overload leads to mitochondrial damage and ultimately produces the symptomatic central cores in the diseased muscles (Boncompagni et al., 2009; Brookes et al., 2004). Similarly, the Ca_V_1.1e-mediated spontaneous calcium sparklets, or the increased calcium influx after store depletion, might overburden the mitochondrial calcium handling capacity and cause their loss in *Ca_V_1.1^ΔE29/ΔE29^* muscles. In fact, both calcium sparks and store-operated calcium currents have previously been implicated in the pathophysiology of muscular dystrophy (Goonasekera et al., 2014; Weisleder and Ma, 2006). Thus, the mitochondrial damage observed in *Ca_V_1.1^ΔE29/ΔE29^* mice might precede the appearance of histopathological and clinical features of DM1.

We conclude that, by itself, missplicing of exon 29 in *Ca_V_1.1* is not sufficient to reproduce the full spectrum of DM1 symptoms in mice. As the disease phenotype in dystrophy and myotonia mouse models is often less severe than in human disease, this does not preclude the possibility that missplicing of Ca_V_1.1 makes a notable contribution to the disease in DM1 patients. Furthermore, it is likely that in combination with the splicing defects in other muscle genes involved in calcium handling (*Serca1*) and excitability (*Clcn1*), the mitochondrial damage observed in the *Ca_V_1.1^ΔE29/ΔE29^* mice might be aggravated, and thus contribute to the myopathy (Tang et al., 2012). If so, the use of clinically approved L-type calcium channel blockers to target Ca_V_1.1e currents might be a viable strategy to alleviate the symptoms of DM1 (Benedetti et al., 2015).

In conclusion, the effects of Ca_V_1.1 missplicing in the *Ca_V_1.1^ΔE29/ΔE29^* mice revealed a novel role of increased L-type calcium currents in the dysregulation of muscle fiber type composition. With regard to normal muscle physiology these findings suggest that, during development, skeletal muscles actively suppress L-type calcium currents by alternative splicing of Ca_V_1.1, so as to prevent unrestrained calcium influx during EC coupling from interfering with the second calcium signaling function in regulating muscle fiber type composition. Furthermore, aberrant expression of the developmental Ca_V_1.1e splice variant in mature muscles causes mitochondrial damage, which might contribute to the pathology of DM1.

## MATERIALS AND METHODS

### Mice and animal care

*Ca_V_1.1^ΔE29^* mice were generated in a C57BL/6N background at Taconic Artemis (Cologne, Germany). All experimental protocols conformed to guidelines of the European Community (86/609/EEC) and were approved by the Austrian Ministry of Science (BMWF-66.011/0069-II/10b/2010) and by the Institutional Animal Care Committee of the University of Debrecen (22/2011/DE MAB). Genotyping is described in the supplementary Materials and Methods.

### Quantitative RT-PCR

RNA was isolated from muscles of wild-type and *Ca_V_1.1^ΔE29^* mice using the RNeasy Fibrous Tissue Mini Kit (Qiagen). After reverse transcription (SuperScript II reverse transcriptase, Invitrogen) the absolute number of transcripts was assessed by quantitative TaqMan PCR (50 cycles). For primer sequences and experimental details, see the supplementary Materials and Methods and Table S1.

### Microarray

Affymetrix GeneChip analysis of mRNA expression in soleus and EDL muscles of wild-type and *Ca_V_1.1^ΔE29/ΔE29^* mice was performed as detailed in the supplementary Materials and Methods.

### Behavioral experiments

The following tests were performed on 2- and 8-month-old wild-type, heterozygous *Ca_V_1.1^+/ΔE29^* and homozygous *Ca_V_1.1^ΔE29/ΔE29^* mice: home cage activity, voluntary running, Rotarod test, wire hang test, grip force measurements. For details, see the supplementary Materials and Methods.

### Force measurements

Contractile force of isolated EDL and soleus muscles of 3- to 6-month-old mice in response to twitch and tetanic stimulations were recorded with a capacitative mechanoelectric force transducer as described in the supplementary Materials and Methods.

### Single fiber measurements

Single fibers were isolated from FDB muscles and used for voltage-clamp recordings of calcium currents with an Axoclamp 2B amplifier (Axon Instruments), and cytoplasmic calcium signals were recorded using fluorescent calcium indicators and imaging with a Zeiss 5 Live confocal microscope (20× objective). Further experimental details and data analysis are described in the supplementary Materials and Methods.

### Immunohistochemistry and SDH activity assay

Cryosections of EDL and soleus muscles from 6-month-old wild-type and *Ca_V_1.1^ΔE29/ΔE29^* mice were immunostained with antibodies against specific myosin heavy chain isoforms (Developmental Studies Hybridoma Bank, University of Iowa, Iowa, USA). Histochemical staining of SDH activity was performed with succinic acid and nitroblue tetrazolium for 1 h. For information on the antibodies used and further details of experimental and quantification procedures, see the supplementary Materials and Methods and Table S2.

### Western blot and calcineurin assay

Cytoplasmic and nuclear protein fractions were prepared from 6-month-old wild-type and *Ca_V_1.1^ΔE29/ΔE29^* mice and separated on 6-10% bis-Tris gels for western blotting. Calcineurin activity was determined using a Calcineurin Phosphatase Activity Colorimetric Assay (Abcam) according to the manufacturer's instructions. For antibodies and experimental details, see the supplementary Materials and Methods.

### Electron microscopy

Tissue processing for transmission electron microscopy of EDL and soleus muscles, and the analysis of mitochondria, were performed as detailed in the supplementary Materials and Methods.

### Statistical analysis

A two-way ANOVA with Bonferroni post-hoc test was used for home cage activity and fiber type analysis. One-way ANOVA was used for the other behavioral tests. Student's *t*-test and Mann–Whitney U-test were used to calculate the statistical significance for fiber type and contractile properties analysis. *N* represents the number of animals. Data are presented as mean±s.e.m. The statistical analysis was performed with GraphPad Prism. For further details see the supplementary Materials and Methods.
